# Supplementary material for: The Roles of the PSEUDO-RESPONSE REGULATORs in Circadian Clock and Flowering Time in Medicago truncatula
Source: Int J Mol Sci. 2023 Nov 28;24(23):16834. doi: 10.3390/ijms242316834 (PMC10706769; doi:10.3390/ijms242316834)
Supplement: Supplementary file 1 [file ijms-24-16834-s001.zip › Supplemental Figures S1-S3.pdf]

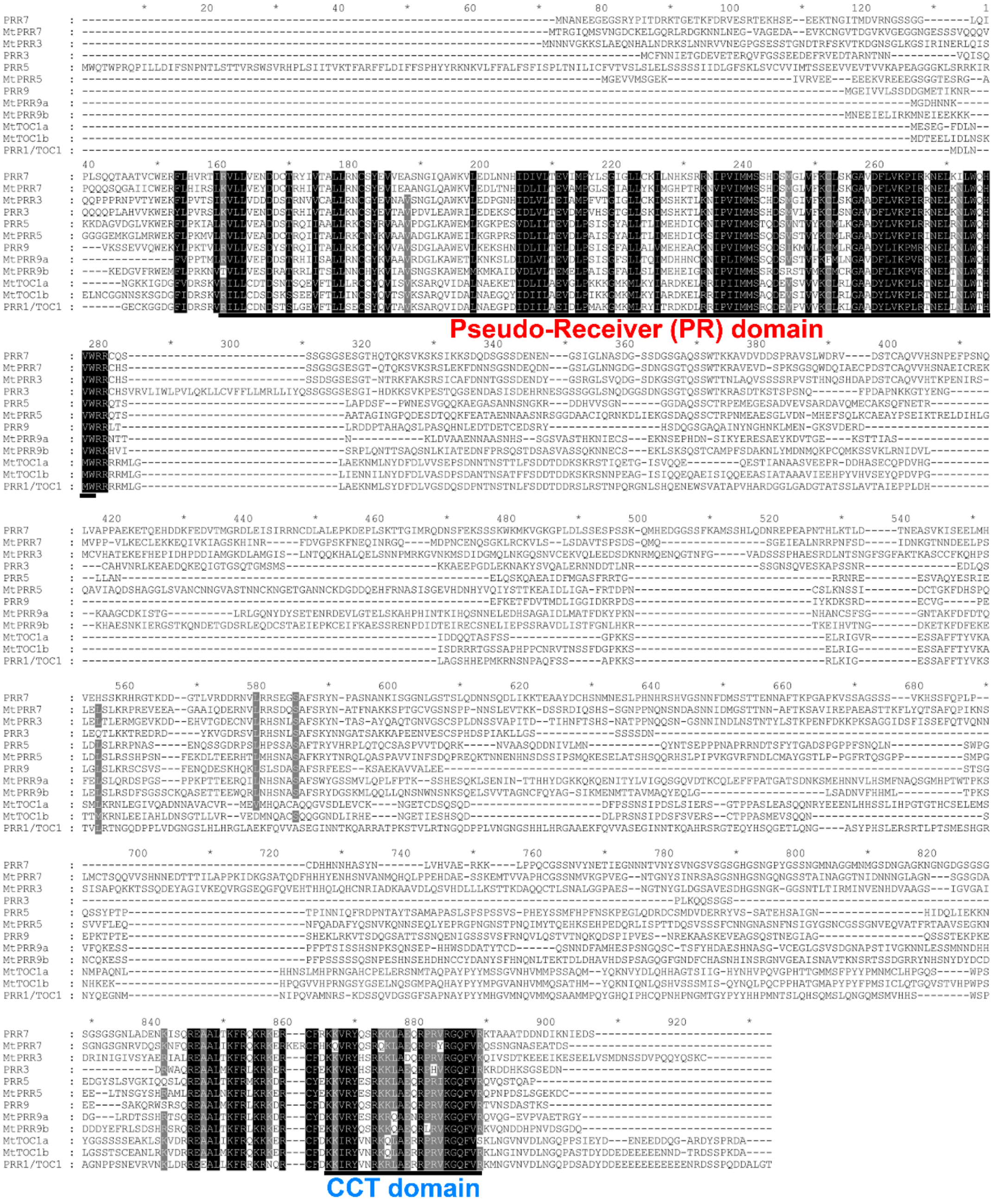


**Figure S1.** Amino acids alignment for PRR proteins in *Medicago truncatula* and *Arabidopsis thaliana*. The PR domain and CTT domain were marked in lines.

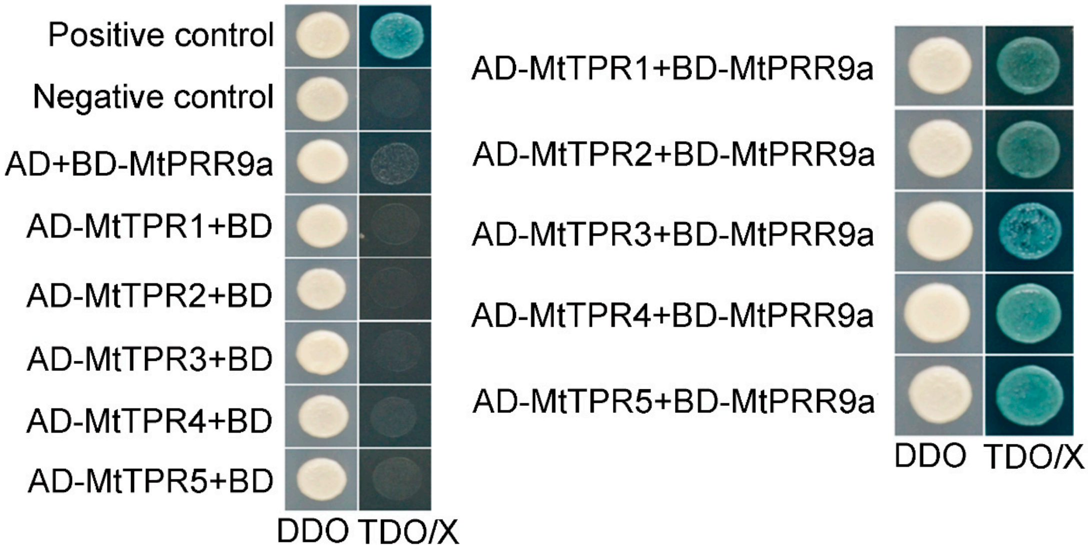


**Figure S2.** Identification of the interactions between MtPRR9a and MtTPR1-5 by Y2H. All transformants can grow on DDO medium. Yeast colonies that were able to grow on TDO with X-α-Gal and displayed blue coloration confirmed the protein-protein interaction.

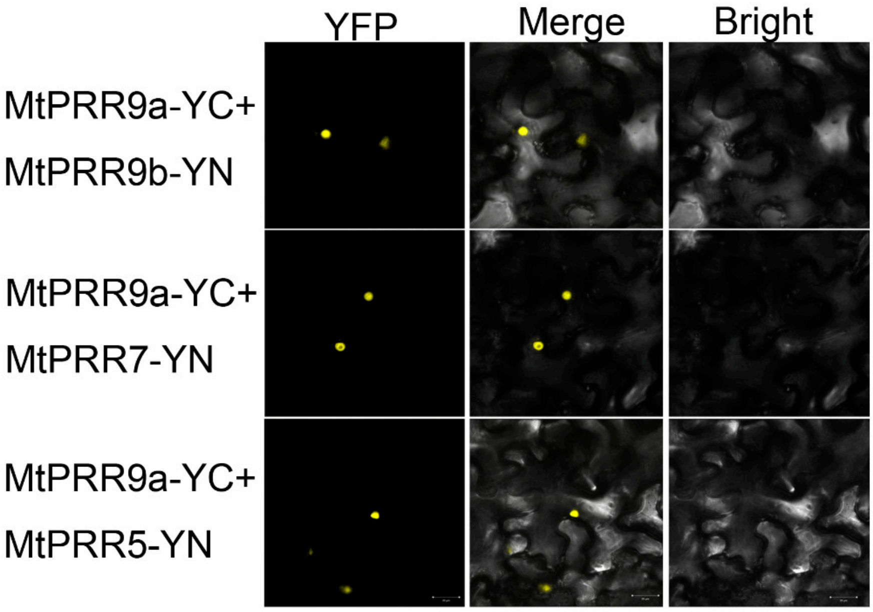


**Figure S3.** BiFC assays indicated that the MtPRR9a interacted with MtPRR9b, 7 and 5 in tobacco leaves.
